# Convolutional Virtual Electric Field for Image Segmentation Using Active Contours

**DOI:** 10.1371/journal.pone.0110032

**Published:** 2014-10-31

**Authors:** Yuanquan Wang, Ce Zhu, Jiawan Zhang, Yuden Jian

**Affiliations:** 1 School of Computer Science, Tianjin University of Technology, Tianjin, China; 2 School of Electronic Engineering, University of Electronic Science and Technology of China, Chengdu, China; 3 School of Software Engineering, Tianjin University, Tianjin, China; 4 School of Computer Science, Beijing Institute of Technology, Beijing, China; Beijing University, China

## Abstract

Gradient vector flow (GVF) is an effective external force for active contours; however, it suffers from heavy computation load. The virtual electric field (VEF) model, which can be implemented in real time using fast Fourier transform (FFT), has been proposed later as a remedy for the GVF model. In this work, we present an extension of the VEF model, which is referred to as CONvolutional Virtual Electric Field, CONVEF for short. This proposed CONVEF model takes the VEF model as a convolution operation and employs a modified distance in the convolution kernel. The CONVEF model is also closely related to the vector field convolution (VFC) model. Compared with the GVF, VEF and VFC models, the CONVEF model possesses not only some desirable properties of these models, such as enlarged capture range, u-shape concavity convergence, subject contour convergence and initialization insensitivity, but also some other interesting properties such as G-shape concavity convergence, neighboring objects separation, and noise suppression and simultaneously weak edge preserving. Meanwhile, the CONVEF model can also be implemented in real-time by using FFT. Experimental results illustrate these advantages of the CONVEF model on both synthetic and natural images.

## Introduction

Image segmentation aims at partitioning the input image into a finite number of disjoint regions, which share certain consistent properties such as intensity and texture. Active contour, or snake model, has been one of the most influential variational models for image segmentation since its debut in 1988 [1]. The basic idea behind the snake model is that an elastic curve **c**(s) = [x(s),y(s)], 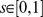, defined in the image domain, deforms to minimize the following energy functional
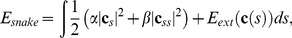
(1)where **c**_s_(*s*) and **c**_ss_(*s*) are the first and second derivatives of **c**(s) with respect to *s* and positively weighted by *α* and *β,* respectively. E_ext_(**c**(*s*)) is the image potential. Using the calculus of variations, the Euler equation to minimize E_snake_ is
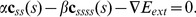
(2)This can be considered as a force balance equation,
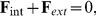
(3)where **F**_int_ = *α* **c**_ss_(s)- *β* **c**_ssss_(s) and **F**_ext_ = 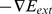. The internal force **F**_int_ keeps the snake contour to be smooth while the external force **F**_ext_ attracts the snake to the desired image features.

Soon after the snake model has been proposed, there has been a flurry of research devoted to the theory and application of this model. Generally speaking, the active contours can be categorized into region-based models [2]–[20] and edge-based models [1], [21]–[28] according to how the image data is utilized. The region-based models usually employ certain region-homogeneity criteria to guide the evolution of the active contours, such as the local region descriptors in [17]–[20] and histogram in [10]–[12]. The advantages of region-based models include robustness to noise and weak edge, and insensitivity to initial contour. Edge-based models utilize the image edge map to stop the evolution of the contour, as a result, the active contours follow high gradient to extract object boundaries and are effective only when the contrast between foreground and background is high. The edge-based snake is an active topic in the computer vision community [22] [23] [27] [28] and we focus on the edge-based parametric active contour in this study.

Under the framework of edge-based active contours, the typical external force is derived from the gradient of the edge map. Due to its local nature, the gradient based external force is ‘myopic’ and not regular enough, as a result, the snake contour must be initialized around the object boundary. In order to overcome the shortcomings, Xu and Prince [29], [30] proposed the gradient vector flow (GVF) external force which largely solved the problem of limited capture range. Although there are many improved works on the GVF [31]–[43], the high computation load of the GVF creates an obstacle for real-time applications. In order to address this issue, several algorithms have been proposed to accelerate the convergence [44], [45]. Park and Chung [46] and Yuan and Lu [47] independently proposed the virtual electric field (VEF) external force, in which each pixel is considered as a static charge. By leveraging fast Fourier transform (FFT), the VEF can be implemented in real time and maintains other desirable properties such as large capture range and u-shape concavity convergence. Recently, the vector field convolution (VFC) model is proposed [48] by convolving the image edge map with a vector field kernel. As stated in [49], the VFC has superior noise robustness and lower computational cost than the GVF model. Very recently, a hybrid structural and texture distinctiveness vector field convolution (STVFC) approach is proposed, where the texture distinctiveness is employed to automatically initialize the snake and to get a better edge map for texture segmentation [50].

In this work, we propose an extension of the VEF model by using modified distance in the convolution kernel. We refer to this extension as CONvolutional Virtual Electric Field, CONVEF for short. As we will point out in section 3, the CONVEF model can also be considered as an extension of the VFC model. In contrast to the STVFC model which focuses on initialization and on better edge map using texture distinctiveness [50], the proposed CONVEF model aims at designing a better convolution kernel. This CONVEF model maintains the common properties of the GVF-like external force, such as enlarging capture range, u-shape concavity convergence, subject contour convergence and initialization insensitivity. What's more, the CONVEF model is more effective on suppressing noise than the GVF, VEF and VFC models and possesses other interesting properties such as G-shape concavity convergence and neighboring objects separation, which are not mentioned in VEF [46] and VFC [48] models. Meanwhile, the CONVEF model can also be implemented in real-time by using FFT. The basic idea of the CONVEF is presented in [51] and has been recently integrated into the anisotropic diffusions for image denoising [52]. Compared with [51], we explore the VFC model with Gaussian-like magnitude and analyze the performance of the VFC model in detail; we further present theoretical analysis of the CONVEF model, and have extended the experimental results.

The remainder of this paper is organized as follows. In Section 2, a brief review of the GVF, VEF and VFC models is presented. In Section 3, the behavior of noise suppression of the GVF, VEF and VFC models is analyzed first and then the proposed CONVEF model is presented. Section 4 reports the experimental results. Conclusions are drawn in Section 5.

## Backgrounds: From GVF to VEF and VFC

### GVF: Gradient Vector Flow

The typical shortcomings of the external force using the gradient vector of the edge map of a given image include limited capture range and poor convergence to concavities [29]. In order to solve these problems, Xu and Prince [29] proposed the gradient vector flow (GVF) model to replace F_ext_ = 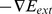 in (3). The GVF is a vector field 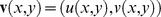 obtained by minimizing the following energy functional [29]:
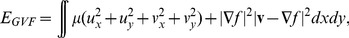
(4)where *f* is the edge map of an image, *μ* is a regularization parameter. Using the calculus of variations, the Euler equation to minimize 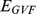 reads:
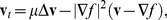
(5)where Δ is the Laplacian operator.

### VEF: Virtual Electric Field

In order to overcome the heavy computation load of GVF, Park and Chung proposed the virtual electric field (VEF) model [46]. In this model each pixel in the image is considered as a virtual electric charge and the virtual electric field at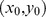, which is created by all other electric charges in region D enveloping 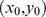, is given by
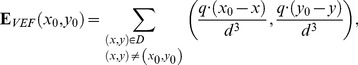
(6)where 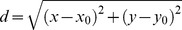, D = {(x,y)| |x-x_0_|≤t, |y-y_0_|≤t}, and 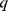 is defined as the magnitude of the edge map of an image. This VEF can be implemented in real time by using FFT since the 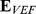 can be rewritten in convolution form as follows,
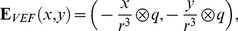
(7)where 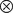 denotes convolution operation and 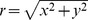 is defined in region R = {(x,y)|−t≤x≤t, −t≤y≤t}. The name VEF is coined in [46], however, the introduction of electric field into active contours could date back to the work by Yue et al [53] where they stated “*we determine E_image_ by following the concept of electric potential energy of an electric charge. Each edge pixel is treated as an electric charge, and the image energy is considered as the contribution from all the electric charges.*” The gradient of the potential energy is just the virtual electric field. This VEF can serve as an alternative to GVF, not only for active contour, but also for other applications such as extraction of curve skeleton [54] and finding symmetry axes [55].

In addition, it is also interesting to mention there is another physical phenomenon being utilized to design the external force for active contours, i.e., universal gravitation [56]. Under this framework, each pixel is a celestial body, between any two single bodies with mass, there exists attractive force acting on each other, which is proportional to their mass product and inversely proportional to the distance between their mass centers. Based on this concept, the gravitation energy field takes the following form [56],
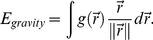
(8)Comparing (8) with (7), if 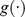 is also defined as the magnitude of the edge map, the VEF would be, to some extent, equivalent to this universal gravitation based external force.

### VFC: Vector Field Convolution

Lately, Li and Acton [48] proposed the VFC external force by convolving the image edge map with a vector field kernel, 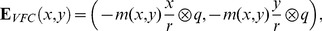
(9)where *r* and *q* possess the same meanings as in (7), and 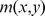
is the magnitude function of vector 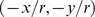
at 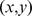 and the authors proposed two types of magnitude function as follows,
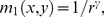
(10)

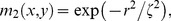
(11)where *γ* and ζ are positive parameters to control the decrease, as the signal-to-noise (SNR) is decreased, *γ* (or ζ) should be decreased (or increased). The authors have already mentioned the equivalence between the VFC using 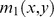 and the universal gravitation based external force. It is also clear that the VFC using 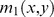 is equivalent to the VEF in (7) if the parameter *γ* in (10) is 2, and therefore, the VFC can be considered as a direct extension of the VEF model.

## The CONVEF Model

### Analysis of the VFC,GVF and VEF Models for Noise Suppression

**Figure 1:**
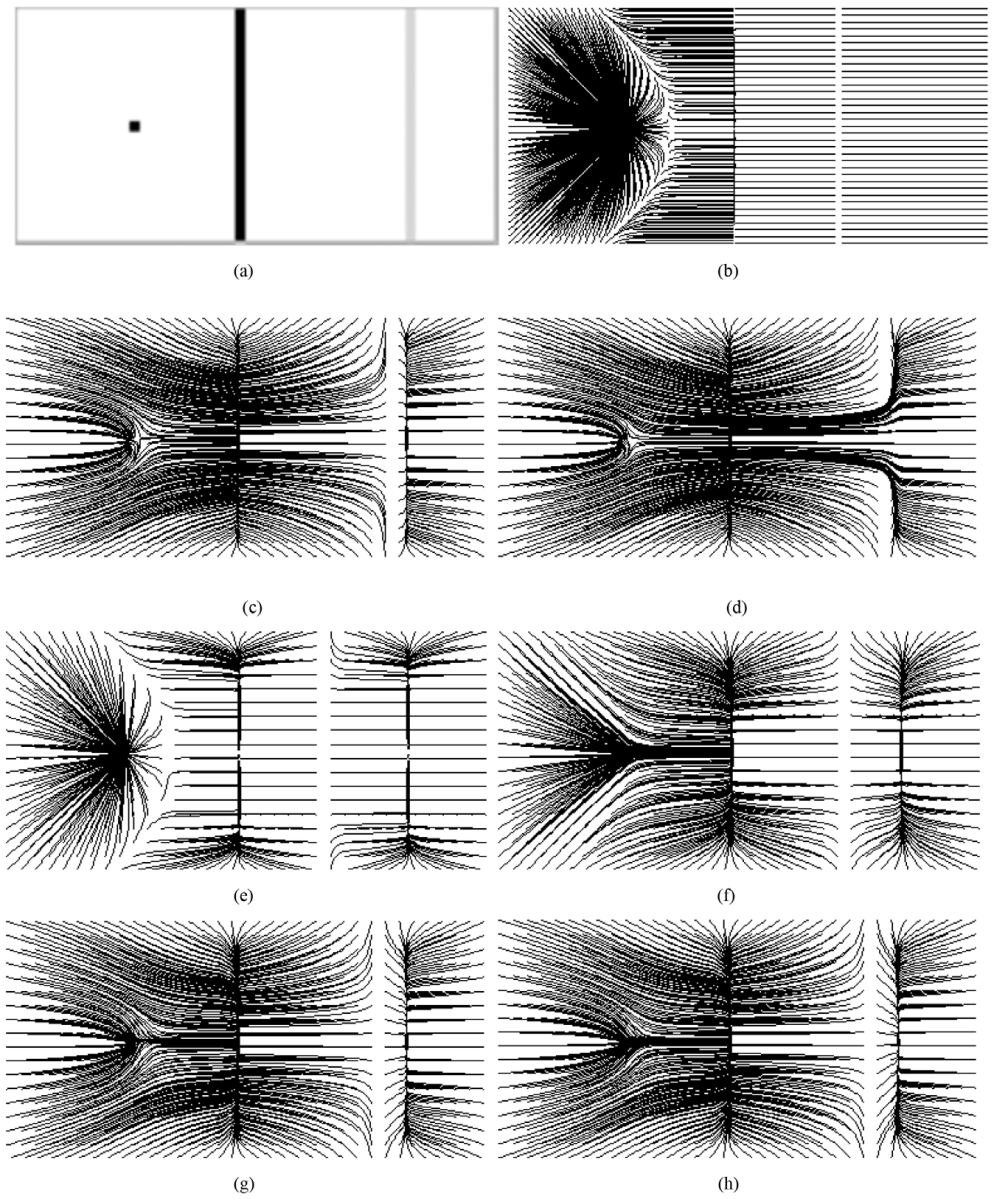
Results on a synthetic image. (a) Synthetic edge map containing an impulse, a strong edge and a weak one; streamlines generated from (b) GVF using *μ = 0.2,#iteration = 200*, (c) VFC using m_1_ with 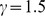, (d) VFC using m_1_ with 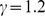, (e) VFC using m_2_ with 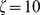, (f) VFC using m_2_ with 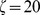; (g) CONVEF with *n* = 1.0, *h* = 20.0, (h) CONVEF with *n* = 1.0, *h* = 25.0.

For the VFC model using 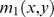, the authors encouraged decreasing *γ* to suppress noise, therefore, 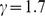 is employed for all the experiments and excellent performance over GVF on noise suppression has been exemplified in [48]. As pointed out in [57], “*a significant advantage in using the VFC force as opposed to standard formulations of external forces or more sophisticated formulations such as the gradient vector flow field (GVF) is that the VFC force is robust to spurious edges and noise in the image and provides a large capture range*.” However, further studies show that the VFC model would also smooth away weak edges while suppressing noise. Fig. 1 shows an example similar to that in Fig. 3 in [48]. There are an impulse, a strong edge and a weak edge in this synthetic image where black is zero and white is unitary. The magnitudes of the strong edge and the impulse are zero and that of the weak edge is 0.85. The streamlines generated from the VFC using 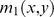 with 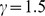 and 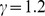 are shown in Figs.1 (c) and (d), respectively, where it can be observed that the force generated from the impulse always plays an important role in the left part of the strong edge even though the weak edge is overwhelmed in Fig. 1 (d). Similar observations also occurred in [48]. Therefore, there is a dilemma for the VFC snake to eliminate noise and preserve weak edges simultaneously. The streamlines of the VFC model using 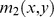 with 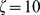 and 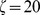 are shown in Figs.1 (e) and (f), respectively. Although the result in Fig. 1(f) is satisfactory, the VFC using 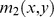 fails when the noise distribution is more complicated. The streamlines of the GVF model is shown in Fig. 1 (b), it is also obvious that the impulse noise dominates the left part of the GVF field. Since VEF is equal to the VFC using 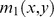 with 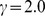, it is clear that the VEF cannot overwhelm the impulse noise as well.

**Figure 2:**
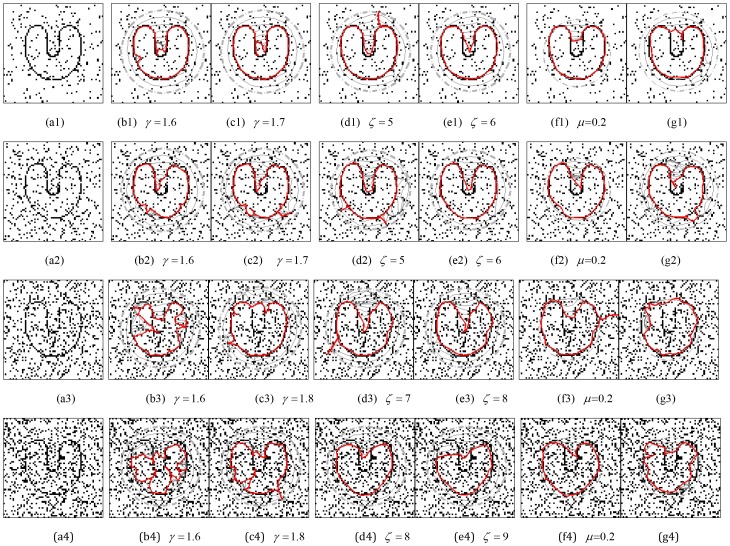
Segmentation results using the VFC, GVF and VEF snakes. The noisy images in Column (a) are coined by using MATLAB function *imnoise* with different noise level. Columns (b) and (c) are the results of VFC using m_1_(x,y), those in columns (d) and (e) are based on m_2_(x,y), those in column (f) are the results of GVF snake with 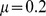, #iteration = 200 for GVF, and those in column (g) are the results of VEF snake. The noisy images are smoothed using Gaussian filter of standard deviation 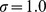 to calculate the GVF field, and the initial contours are very close to the u-shapes, especially in the 3^rd^ and 4^th^ rows, for the GVF and VEF snakes. It is obvious that all the results are not satisfactory because of edge leakage, being trapped in local minima and failure to converge to concavities.

There are other demonstrations in Fig. 2. Resembling the noisy image in Fig. 8 in [48], the impulse noise is added to the U-shape image by using MATLAB function *imnoise(U, 'salt&pepper', Var)* with *Var* varying from 0.1 to 0.4 with step 0.1 in from the first row to the fourth row in Fig. 2, respectively. The goal in these examples is to extract the U-shape object from the noisy images. There are three handicaps in achieving this goal: (1) the evolving contour would get trapped in local minima arising from noise; (2) since the object boundary may be broken by noise, the evolving contour would leak out; (3) it is difficult for the contour to converge to the noisy concavity. Since the noises in the present examples are much heavier than those in [48], it is more difficult for the snakes to converge correctly to the concavities. We also let the noisy image stay intact as done in [48]. The results of VFC snake using 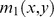 are shown in columns (b) and (c), those using 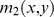 are in columns (d) and (e). The corresponding values of the parameters in 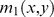 and 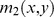,i.e., *γ* and ζ, respectively, are shown in the subcaption of each subfigure. These values are chosen so that they are *justifiable*, for example, in Fig. 2(b1), *γ* is 1.6 but the contour leaks out; when *γ* is 1.5 the leakage is more serious. Therefore, we set *γ* to 1.7 to resist leakage in Fig. 2(c1), however the concavity convergence is poorer than that in Fig. 2(b1) and the contour converges more poorly when *γ* is 1.8. Consequently, the results of 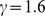 and 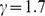 are chosen for demonstration. The values for ζ are also chosen in the same way. The increasing step is 0.1 for *γ* and 1.0 for ζ. We found when the step for ζ is 0.5, there is usually no significant change in the result, for example, when 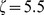, the converged result is similar to that in Fig. 2 (d1) where 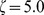, so 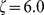 in Fig. 2 (e1). It is obvious that the VFC model using 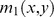 cannot conquer the above mentioned three handicaps at all, the model using 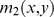 performs slightly better; however, the result are not satisfactory especially in the third and fourth rows in Fig. 2. What's more, we will demonstrate that the VFC model using 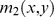 behaves clumsily on G-shape convergence in the next section. The results of the GVF snake are presented in column (f). The noisy images are smoothed using Gaussian filter of standard deviation 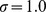 to calculate the GVF field, and the initial contours are very close to the u-shapes, especially in the 3rd and 4th rows, however, the results are far from satisfactory. Since the VEF is equal to the VFC using 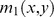 with 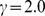, the results in column (g) are not better, if not worse, than those in column (c) where *γ* is 1.7 or 1.8.

### The Proposed CONVEF Model

In order to overcome the dilemma of eliminating noise and simultaneously preserving weak edge encountered by the VFC and VEF models, we propose a further extension of the VEF model by modifying the distance metric. We depart from the concept of electric potential. Following the definitions in (6), the virtual electric potential (VEP) at 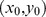 is given by
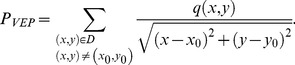
(12)This is a weighted sum and can be rewritten via convolution due to the fact that the weight 
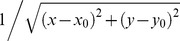
 is not correlated with the signal *q*(x,y). Therefore, the potential takes the following form
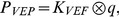
(13)where 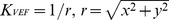, and the associated electric field which is the gradient of the electric potential can also be rewritten via convolution as in (7). If one neglects the physical nature of (12) and take (13) just as a convolution operation, some other convolution kernels can be employed in (13). These new kernels may not necessarily bear any physical meanings any longer; however, they would make VEF more powerful and flexible than the original version. We refer to this convolution based version as CONvolutional Virtual Electric Field, CONVEF for short and to the snake models with CONVEF external force as CONVEF snakes.

We present here one practically effective kernel by modifying the distance metric in 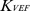. One nonnegative factor *h* is introduced into *r* so that 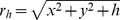, and the power of *r* is relaxed from 1 to a certain positive real number *n*, therefore, the proposed kernel is formulated as
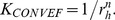
(14)and the corresponding VEP is
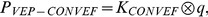
(15)and the gradient of the VEP, i.e., the CONVEF field, is given by
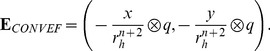
(16)It is obvious, when *h* = 0 and *n* = 1, the CONVEF model reduces to the VEF model. This modification of *r* makes the CONVEF more powerful than the VEF. On one hand, the factor *h* plays a role analogous to scale space filtering, the larger the value of *h*, the greater the smoothing effect on the results, as a result of which the CONVEF snakes would be more robust to noise. In general, the value of *h* should be increased as the signal-to-noise ratio (SNR) is decreased. On the other hand, the larger the value of *n*, the faster the potential decays with distance and vice versa, this property allows the CONVEF snakes to preserve edges and to tell apart two closely-neighboring objects with large *n* (larger than 1, taking the VEF model as reference) and to dive into C-shape concavities with small *n*. Note that we neglect a constant *n* outside the bracket in (16).

**Figure 3:**
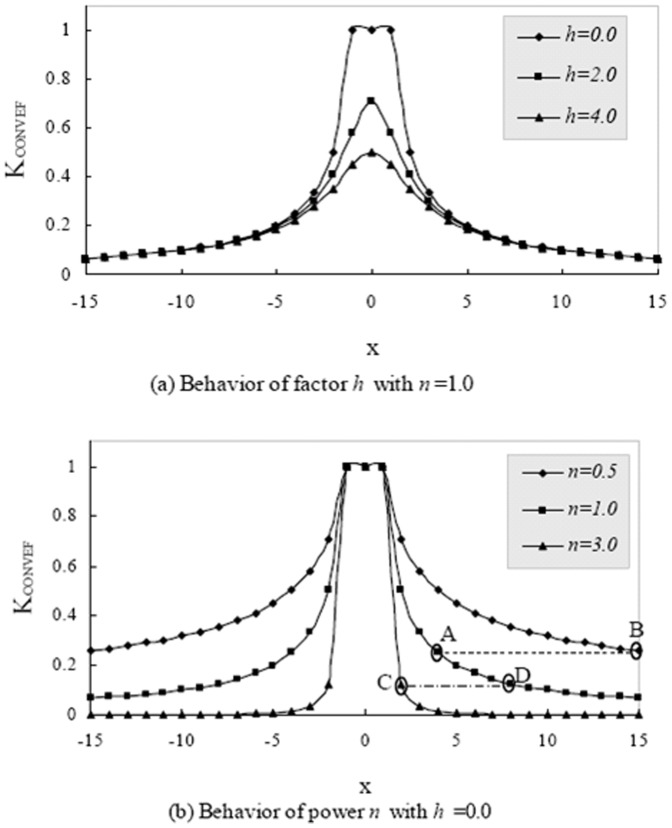
Analysis of the behaviors of *h* and *n* in 1D case.

In order to better understand the behaviors of *h* and *n*, we plot the kernel 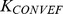 in 1D case with different *h* and *n* in Fig. 3. It can be seen from Fig. 3(a) that, the larger the value of *h*, the smaller the value of 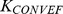 at points near x = 0, but almost unchanged at points far from x = 0. Recall that the convolution in (15) is a weighted sum, this property means larger *h* weighs less information from nearby points in the sum, consequently, the potential 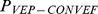 is much smooth and its gradient, the electric field, is much regular. Note that 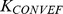 is not defined at x = 0 when 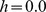, we set 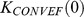 = 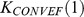 for the purpose of exhibition. Similar strategy is employed in Fig. 3 (b) where it can be observed that the larger the value of *n*, the faster 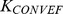 decays with distance. For example, although point A is 4 far from x = 0 while B is 15, due to varying *n*, the values of 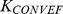 at point A and B are almost identical, it seems as if the point B is as near to x = 0 as point A in terms of the values of 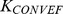; Similar results can be observed for points C and D and it seems as if the point C is as far as D from x = 0. As a result, if one wants to separate two closely-neighboring objects or preserve edges, large *n* can be employed such that nearby points are less weighted as if they are far away; on the other hand, if concavity is too deep, small *n* can be employed to weigh relatively more on faraway points as if they are nearby. Although the CONVEF model is derived from the VEF model, it can also be taken as an extension of the VFC model, since the VFC in (10) is a special case of the CONVEF with 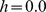 in form. Certainly, the significant difference between the two models is that, the VFC model encourages decreasing *γ* for noise suppression while the CONVEF model employs factor *h* for noise suppression and encourages large *n* for edge preserving and small *n* for C-shape concavity convergence. These factors make the CONVEF model more effective and more versatile than the VFC model. To note, the power of 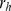 in CONVEF is larger than 2 whereas that of *r* in VFC is just larger than 1 according to their different derivations.

## Experimental Results

In this section, we first show the computation efficiency of the CONVEF model and demonstrate the performance of the CONVEF model on noise suppression and weak edge preserving by comparing with the VFC, VEF and GVF models, then illustrate other interesting properties of the CONVEF model, such as blob-like concavity convergence and neighboring objects separation, on both synthetic and natural images. The parameters for all snakes in our experiments are 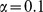, 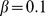, time step 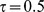, 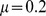 for all GVF, and the size of convolution kernel is the same as that of the image unless otherwise stated.

### Computational Cost

**Figure 4:**
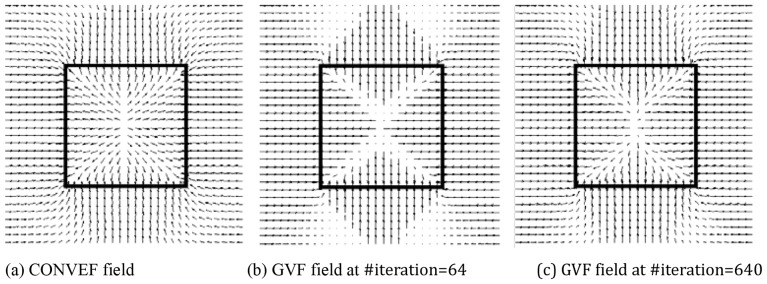
An example of the line-drawing image with N = 128 used for computational cost comparison. It is clear that the GVF field in (b) is unmatured at iteration 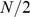, so, the runtime reported in Table 1 for GVF is underestimated.

In order to demonstrate the computation efficiency of the CONVEF model, we coined line-drawing images of dimension 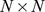 to calculate the CONVEF and GVF fields. Since the VEF, VFC and CONVEF models can all be calculated using FFT, their computational costs depend mainly on the size of the convolution kernel. Therefore, we just compare the runtime of the CONVEF model with that of the GVF model. The convolution kernel size for CONVEF is 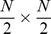, and the iteration number for GVF is 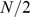. The results with different *N* are reported in Table 1, from which one can see that the CONVEF is about 10 to 400 times faster than the GVF since there are two partial differential equations for the GVF to be solved iteratively on the entire image. Fig. 4 shows an example of the line-drawing image with N = 128. It is clear that the GVF field in Fig. 4(b) is unmatured at iteration 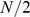, so, the runtime reported in Table 1 for GVF is underestimated. This experiment was conducted using MATLAB 2010 on a Thinkpad T60 notebook with 1.83 GHz CPU, 2.5 GB RAM.

**Table 1:** Comparison of the runtime of GVF and CONVEF for an 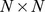 image.

Runtime(second)	N					
	2048	1024	512	256	128	64
GVF	9739.5	1188.8	116.2	12.3	0.93	0.22
CONVEF	24.01	3.89	1.04	0.27	0.05	0.02

### Noise Robustness Using Factor h

To evaluate the noise suppression ability of CONVEF model using *h*, we also calculated the CONVEF field on the synthetic image in Fig. 1. The streamlines generated from the CONVEF with fixed 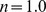, 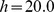 and 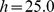 are shown in Figs.1 (f) and (g), respectively. It is clear that the force generated from the strong edge overwhelms that from the impulse while the weak edge is preserved in both cases. The reason behind the success of CONVEF is obvious. Large power of 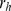 (say, 3 in this experiment) makes 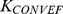 decay fast and the weak edge would be preserved; while large *h*(say, 20 and 25 in this experiment) weighs less information from nearby points, therefore, the impulse is less weighted in generating the force field. Consequently, one can combine the use of large *n* with large *h* to simultaneously suppress noise and preserve weak edge.

**Figure 5:**
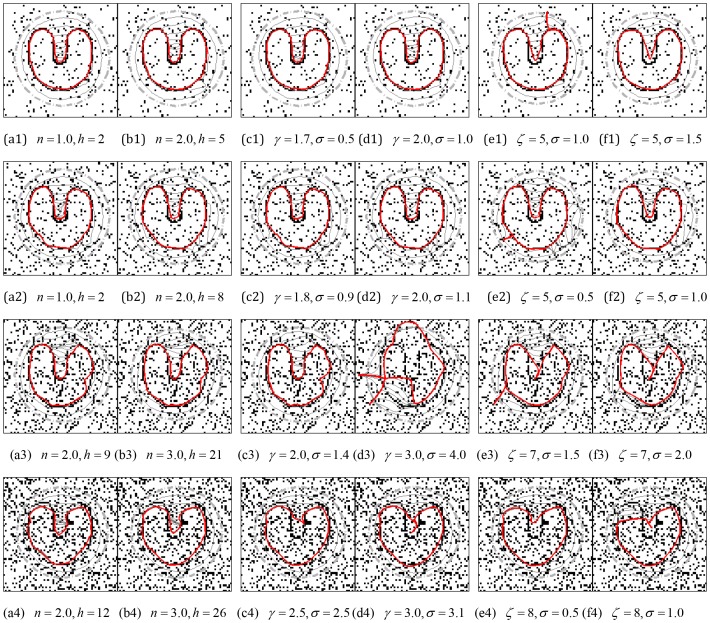
Segmentation results of the noisy images in Fig. 2 using the CONVEF snake and comparisons with the VFC snake. Columns (a) and (b) are the results using CONVEF snakes, those in columns (c) and (d) are the results using VFC snakes with m_1_(x,y) and those in columns (e) and (f) are the results using VFC snakes with m_2_(x,y). Before calculating all the VFC fields, the noisy images are preprocessed using a 2D Gaussian function with standard deviation σ. Although the VFC model prefers small *γ* for noise suppression, we increased *γ* to preserve edges in these experiments. The corresponding parameters are listed in the subcaption of each subfigure.

Since the VFC, VEF and GVF snake fails to extract the U shape in the noisy images in Fig. 2, the CONVEF snake is employed for this task. Through the observations in Fig. 1, we can increase the value of *h* to suppress noise and increase the value of *n* to preserve edges. The results of CONVEF snakes at some combinations of *h* and *n* are shown in columns (a) and (b) in Fig. 5. These results are satisfactory even though the noise is very heavy in the fourth row. Although the VFC snake fails to overcome the three handicaps in extracting the U-shape objects in Fig. 2, we propose to presmooth the noisy images using a 2D Gaussian function with standard deviation *σ*,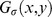. The results of VFC snakes with 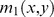 combined with 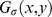 are shown in columns (c) and (d). It is clear this combination would work well when the noise is not very heavy, see the first and second rows in columns (c) and (d). However, when the noise is as heavy as that in the fourth row, it would fail. Although the result in Fig. 5(c3) is fairly good, the result is sensitive to parameters *γ* and *σ*. We have tried many combinations of *γ* and *σ* for the noisy image in the third row, the values of *γ* are {1.8,1.9,2.0,2.5,3.0,4.0}, *σ* varies in a wide range by step 0.1, we found that there are only two combinations, i.e., {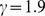, 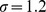}, {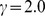, 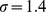}, at which the results are acceptable. However, the CONVEF snake is more robust to the parameters *h* and *n*, for example, when 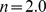, *h* can vary from 9 to 13, at which the results are fairly good. The results of VFC snakes with 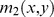 combined with 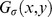 are shown in columns (e) and (f). However, the Gaussian filter didn't help much. The effectiveness of a Gaussian filter is similar to that of increasing ζ in 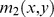. For example, the result in Fig. 5(f2) (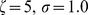) is similar to the result in Fig. 2(e2)(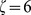).

### Blob-like Concavity Convergence Using Small n

Although the strategy of decreasing the value of *n* in (16) is not a good choice for noise suppression, there may be some other applications in which the CONVEF snake with small *n* plays an important role. The problem associated with convergence to blob-like concavity is one such application. The problem associated with convergence to U-shape has been intensively studied using the GVF, VEF and VFC models. However, it is seldom reported for S-shape, 3-shape, C-shape, and G-shape. The S-shape and 3-shape are slightly more difficult to be extracted than the U-shape since they can be considered as two U-shapes assembled in different ways. However, the C-shape and G shape are more difficult than the U-Shape. The difference between C-shape concavity and U-shape concavity is that the C-shape is semi-close, while the U-shape is open, and there is orientation rotation in the case of G-shape. It is very easy for the GVF and VEF to form source within concave regions and the vectors around the neck of the concave regions are outward; however, for the CONVEF with small *n*, the faraway points will be weighted more and the force field will be affected by more points around, as a result, the CONVEF field around the neck of the concavity will point inward the concavity.

**Figure 6:**
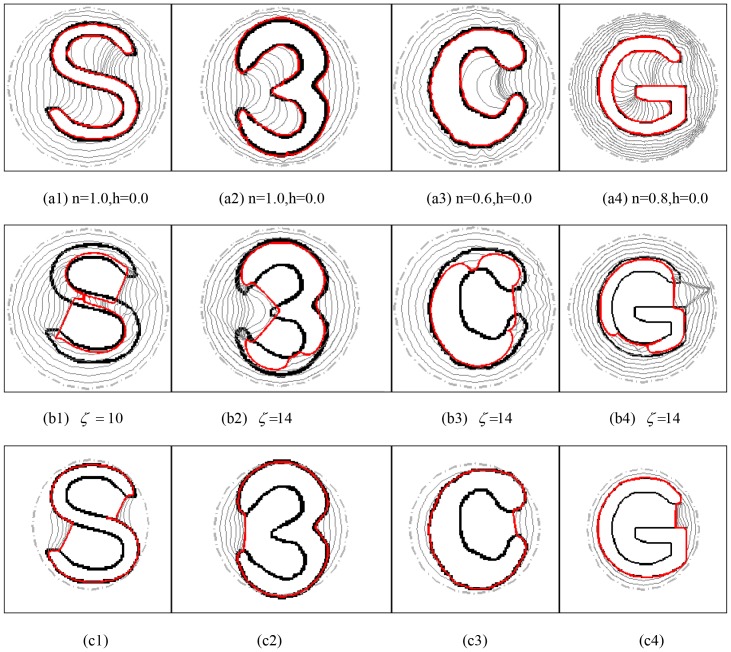
Blob-like concavity convergence of the CONVEF, VFC, and GVF snakes. The results using CONVEF snake are in row (a), and those in rows (b) and (c) are the results using VFC and GVF snakes, respectively. The parameters to calculate the GVF field are 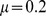, and #iteration = 200, and the other corresponding parameters are listed in the subcaption of each subfigure.

Fig. 6 shows the results of the CONVEF, VFC, and GVF snakes on S-shape, 3-shape, C-shape, as well as G-shape. The results show that the CONVEF snake evolves into the concave region progressively and steadily and locate these blob-like objects correctly. However, the GVF and VFC snakes failed. The success of the CONVEF snake is attributed to weighting more on faraway points with small *n*. The VFC snakes with 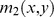 fails to converge to these concavities although the ζ in 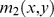 is large enough and boundary leakage occurs. Another observation in row (c) for the GVF snake is that the initial contours are very close to the objects. The reason behind this observation is that the capture range of GVF is not large enough and there are *critical points* [58] which should be outside the initial contours.

### Neighboring Objects Separation Using Large n

**Figure 7:**
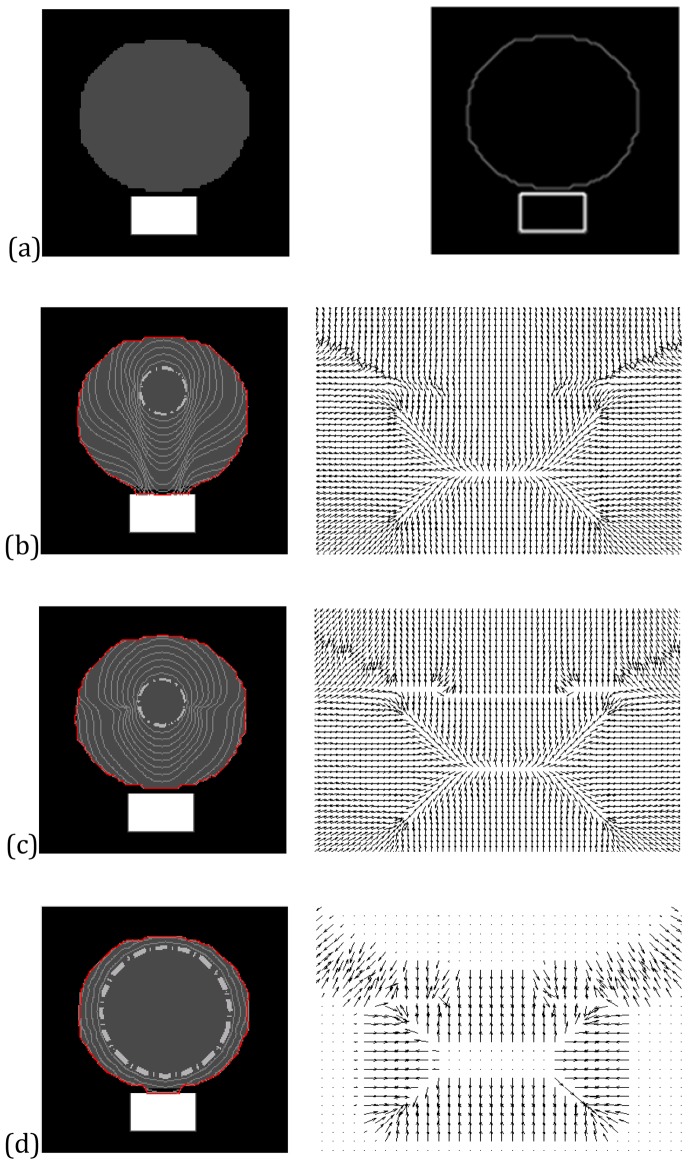
In each panel, from left to right, (a) synthetic image of two objects and its edge map; convergence and the close-up of force field of (b) VEF snake, (c) CONVEF snake with n = 3.0, h = 0.0, (d) VFC snake using m_2_ with 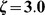
.

Fig. 5 demonstrates the use of large *n* for edge preserving when the CONVEF snake is employed to locate objects. One can also employ large *n* to separate two closely neighboring objects, especially when one edge is weak and the other is strong. In fact, to separate objects is essential to preserve the edge of each object. We demonstrate this particular application using a synthetic image. Fig. 7(a) shows the original image, where there are one gray disk and one white rectangle on the black background and there are just three pixels between two objects. The edge of the disk is weak and that of the rectangle is strong. Fig. 7 (b) shows that the VEF snake moves across the weak edge and sticks to the strong one. Fig. 7 (c) shows the result of the CONVEF snake with 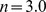 and 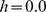. It is clear that the CONVEF snake correctly tells apart these two closely neighboring objects. Fig. 7(d) shows the result of VFC snake using 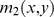 with 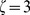. Since the VFC snake prefers large ζ to enlarge the capture range, but ζ is just 3 in this example and the capture range is very low, so the initial contour is close to the disk. However, the snake contour still leaks out to the rectangle.

### Common properties: enlarged capture range, subject contour convergence and initial insensitivity

**Figure 8:**
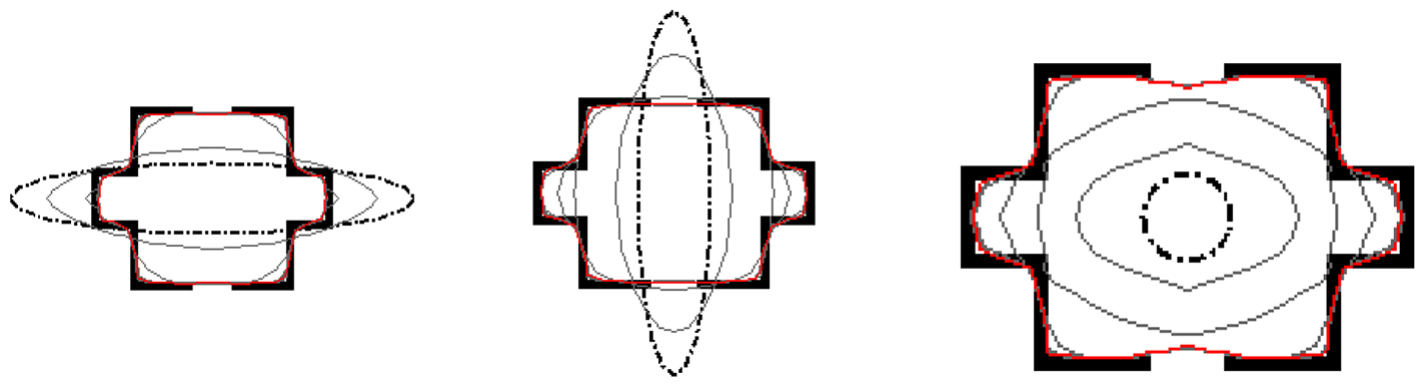
Convergence of the CONVEF snakes with different initial contours.

We utilize the room image, which are also employed in [29] [46] [48], to verify the performance of the CONVEF snake in capture range enlargement, subject contour convergence and insensitivity to initialization. Fig. 8 shows the results on the room image. It can be seen from this experiment that the CONVEF snakes converge to object boundaries and stay on the gaps on the boundaries whenever the initial contour is inside, or outside, or across the boundary. These experiments manifest the CONVEF snake has a large capture range and is insensitive to initialization.

### Real Medical Images

**Figure 9:**
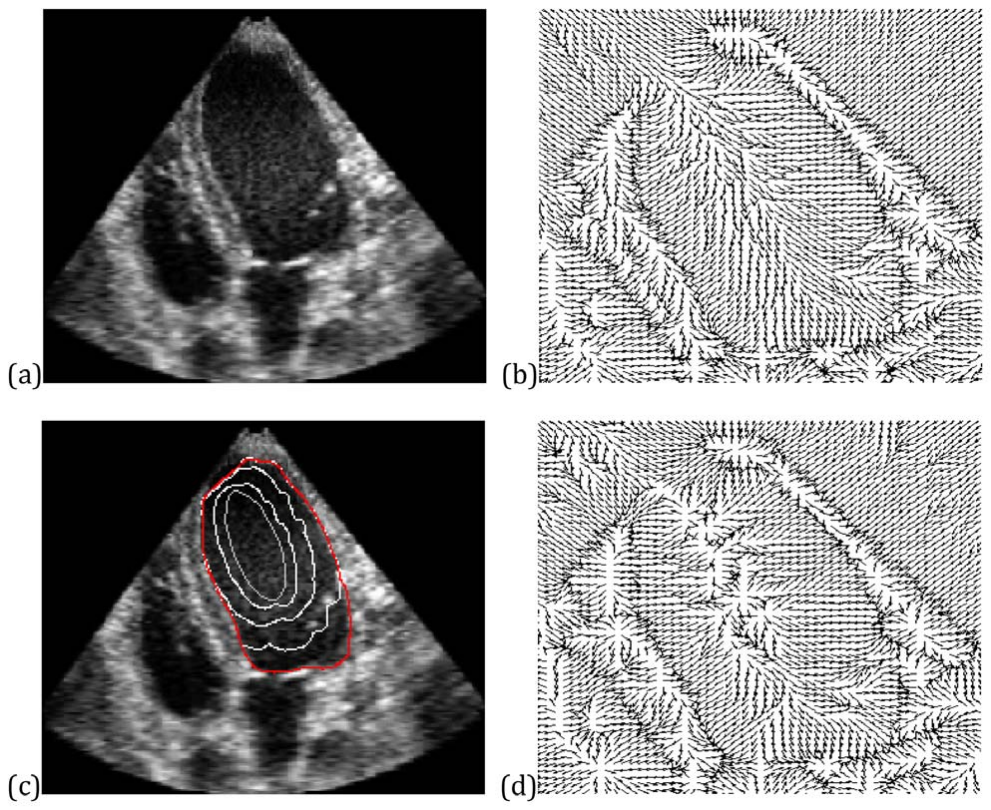
Results on an ultrasound image. (a) Ultrasound heart image, (b) VEF field, (c) convergence of CONVEF snake and (d) its CONVEF field with n = 2.0, h = 10.0.

The CONVEF snake is also applied to real noisy medical images. The first example is an ultrasound heart image with weak edges on the upper-left region. The original image and the VEF field are shown in Figs. 9(a) and (b), respectively. It can be seen from Fig. 9(b) that the VEF field overwhelms the weak edge and flows into the blood pool. It is sure that the VEF snake cannot extract the endocardium correctly whatever is the initial contour. The evolution and the corresponding force field of the CONVEF snake are shown in Figs. 9(c) and (d), respectively. Although the speckle noise is troublesome, the CONVEF field within the blood pool is fairly regular and the CONVEF snake works well. This shows once again that the CONVEF snake provides a superior alternative to the VEF snake.

**Figure 10:**
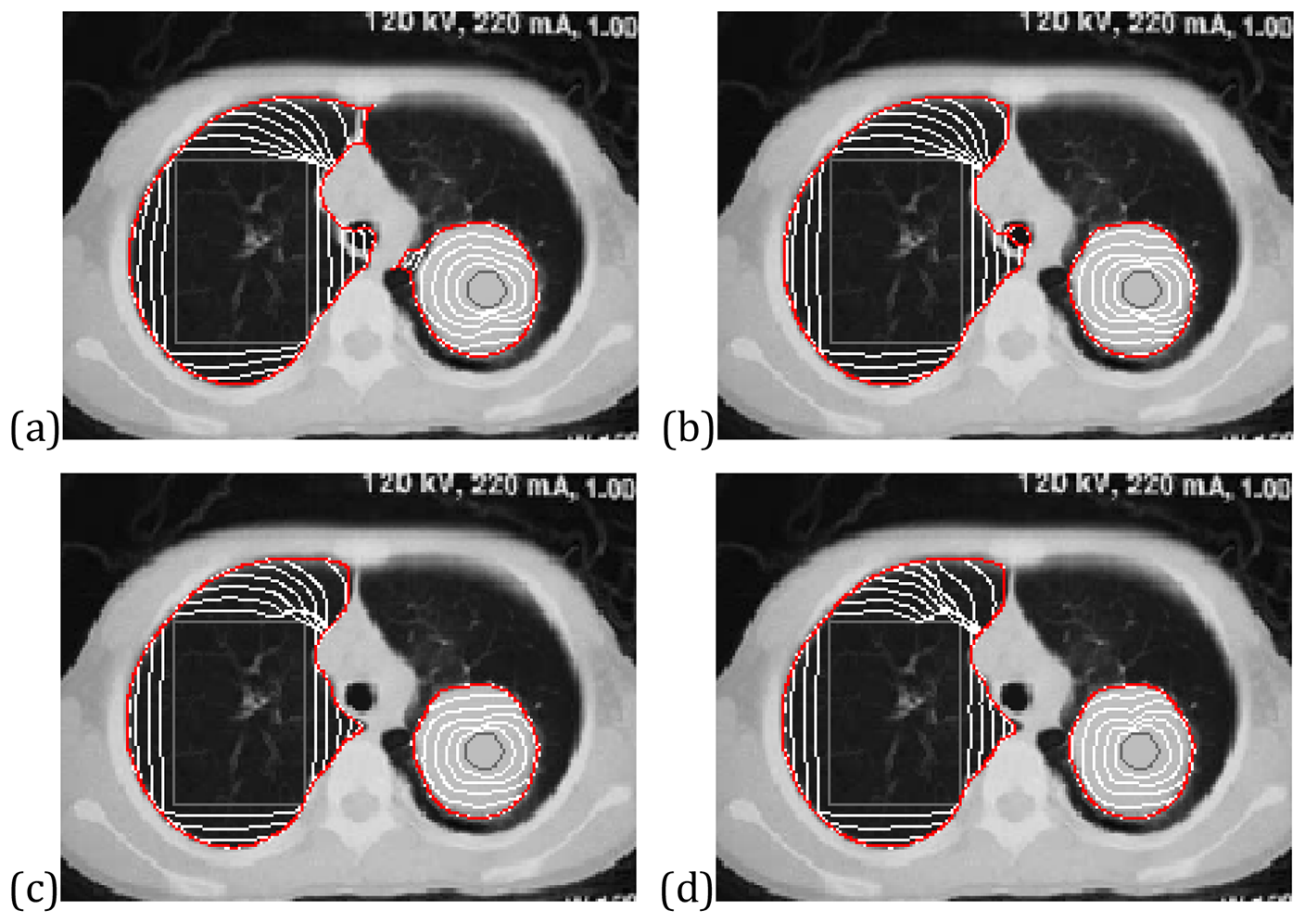
Segmentation of the human lung CT image using (a) VFC snake with 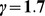, (b) VEF snake, (c) CONVEF snake with n = 1.5, h = 0.0, and (d) CONVEF snake with n = 3.0, h = 5.0.

Fig. 10 shows the segmentation results of the VFC, VEF and CONVEF snakes on a human lung CT image. The purpose of this example is to extract the parenchyma in the left part and the cancer in the right part, and the difficulties reside in the weak edge and closely-neighboring boundaries. The results of VFC and VEF snakes are shown in Figs. 10 (a) and (b), respectively, and the convergent contours of both snakes leak out although the VEF snake behaves much better than the VFC snake. Figs. 10 (c) and (d) show the results of the CONVEF snakes with different parameter settings. Once again this experiment exemplifies the abilities of the CONVEF snake for weak edge preserving and neighboring objects separation.

**Figure 11:**
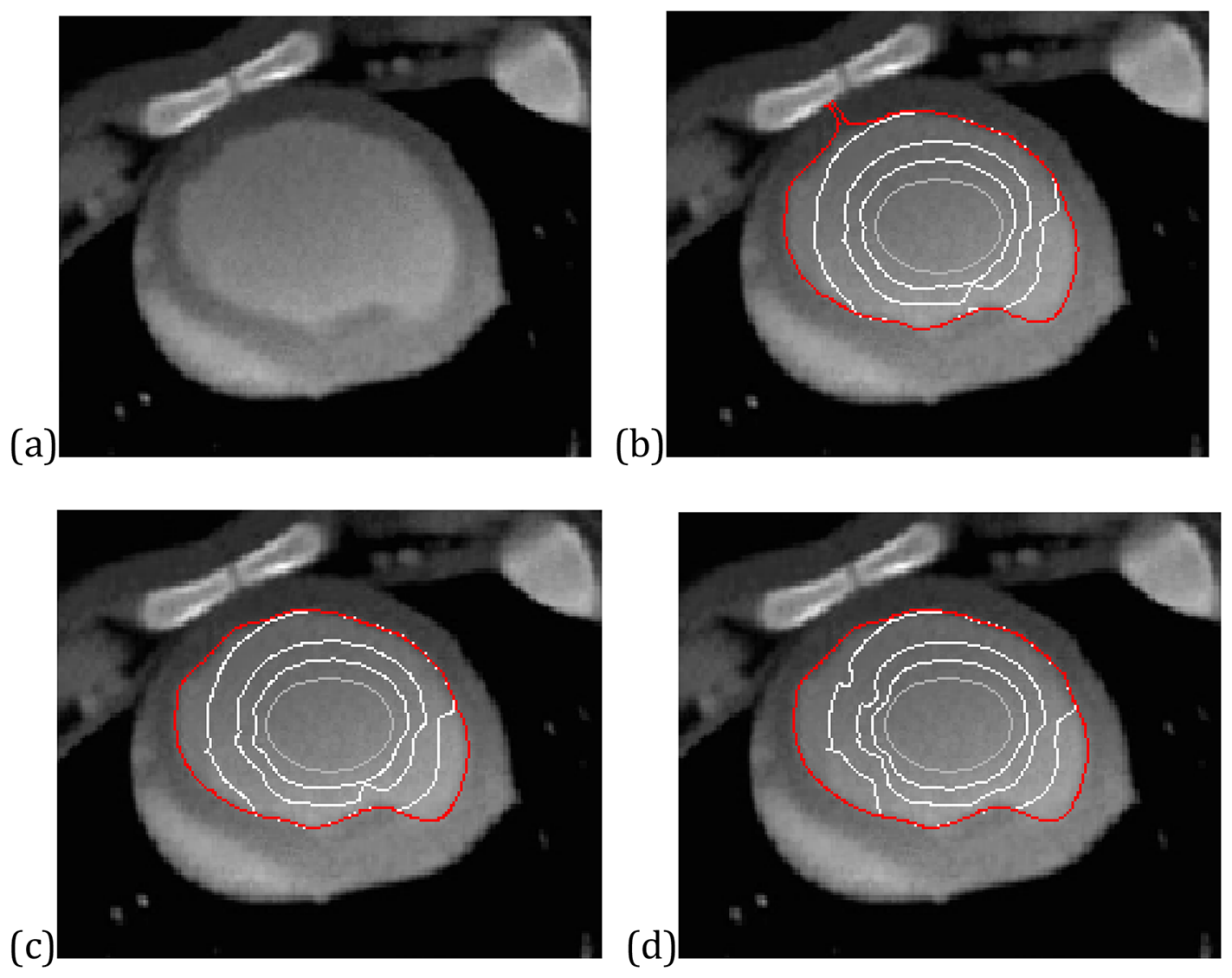
Results on a CT image. (a) Cardiac CT image, convergence of (b) VEF snake, (c) CONVEF snake with n = 1.5, h = 2.0, and (d) CONVEF snake with n = 2.0, h = 6.0.

A third example is a cardiac CT image with inhomogeneity, shown in Fig. 11(a). We also aim at extracting the endocardium of the left ventricle (LV). The difficulty for this task is the strong edges stemming from the bones nearby the LV. Fig. 11(b) shows the evolution of the VEF snake while Figs. 11(c) and (d) show the results of the CONVEF snakes with different parameter settings. These results show that the VEF snake leaks out and moves across the endocardium boundary and sticks to the bones, whereas the CONVEF snakes converge correctly to the boundary of the endocardium.

## Conclusions

In this paper, we have introduced a novel external force for active contours, namely, convolutional virtual electric field (CONVEF). This CONVEF model is derived from the VEF model by designing a more effective convolution kernel. It can also be considered as an extension of the VFC model. The CONVEF snake possesses some desirable properties of the GVF, VEF and VFC snakes, such as large capture range, insensitivity to initialization, and convergence to U-shape concavity and subject contours. Meanwhile, the CONVEF model can also be implemented in real-time by using FFT. What's more, the CONVEF snake behaves much better than the GVF, VEF and VFC snakes in noise suppression, weak edge preserving, blob-like concavity convergence, and neighboring objects separation. These properties of the CONVEF model have been tested on both synthetic and real medical images. It is shown that the CONVEF model can serve as a superior alternative to the GVF, VEF and VFC models. In addition, one can also integrate this CONVEF model into the geometric active contour as done in [34], the texture distinctiveness [50] can also be combined with the CONVEF model, and there may be potential applications of the CONVEF model to extract the curve skeleton [54] and to find axes of symmetry [55]. The ant foraging algorithms [59]–[62] can also be employed to optimize the segmentation results based on active contours, and this is the topic of further research.
